# miRNA regulation of nutrient homeostasis in plants

**DOI:** 10.3389/fpls.2015.00232

**Published:** 2015-04-10

**Authors:** Soumitra Paul, Swapan K. Datta, Karabi Datta

**Affiliations:** Translational Research Laboratory of Transgenic Rice, Department of Botany, University of CalcuttaKolkata, India

**Keywords:** miRNA, plant, micronutrient, macronutrient, transporters, nutrient homeostasis

## Abstract

Small RNAs including micro RNAs (miRNA) play an indispensable role in cell signaling mechanisms. Generally, miRNAs that are 20–24 nucleotides long bind to specific complementary transcripts, attenuating gene expression at the post-transcriptional level or via translational inhibition. In plants, miRNAs have emerged as the principal regulator of various stress responses, including low nutrient availability. It has been reported that miRNAs are vital for maintaining nutrient homeostasis in plants by regulating the expression of transporters that are involved in nutrient uptake and mobilization. The present review highlights the role of various miRNAs in several macro- or micronutrient deficiencies in plants. Understanding the regulation of different transporters by miRNAs will aid in elucidating the underlying molecular signal transduction mechanisms during nutritional stress. Recent findings regarding nutrient related-miRNAs and their gene regulation machinery may delineate a novel platform for improving the nutritional status of cereal grains or crop biofortification programs in the future.

## Introduction

Plants acquire mineral ions from the soil, which they allocate to various compartments following long- or short-distance transport. The underlying molecular mechanisms of uptake, transport, and loading of mineral ions into storage organs depend on the differential expression of various transporters based on mineral availability (Sperotto et al., 2014). The deficiency of mineral ions in the soil elicits signaling responses in plants, and interactions among signaling molecules and transporter or carrier genes can facilitate ion transport (Vigani et al., 2013a, b). If a plant is under nutrient starvation, the information is immediately transmitted to the genetic material via signaling molecules to maintain nutrient homeostasis. The scarcity of nutrients activates transporters or alters the architecture and growth of roots to enhance mineral uptake (Jung and McCouch, 2013). Signaling molecules interact with specific nucleotide sequences of mRNA to alter gene expression.

Small regulatory RNAs are considered the most ubiquitous signaling molecules that regulate post-transcriptional gene expression. These small RNAs bind with specific mRNA that exhibit their perfect complementary bases and attenuate gene expression (Bartel, 2004). In plants, two major types of small RNAs, small interfering RNAs (siRNAs) and microRNAs (miRNAs), are likely associated with the silencing of gene expression. siRNAs, which are small 20- to 24-nucleotide regulatory RNAs, are primarily derived from endogenous genomic regions or exogenously supplied duplexes of nucleic acid, as used in RNAi-mediated gene silencing technology (Jamalkandi and Masoudi-Nejad, 2009; Khraiwesh et al., 2012). Dicer-like enzymes (DCL in *Arabidopsis*) cleave the double-stranded duplex (with hairpin loop) to form short, perfect duplexes, which are loaded onto the RNA-induced silencing complex (RISC). The sense strand of the RISC complex recognizes the target gene or transcript and binds to the perfect complementary nucleotide sequences and regulates the gene expression level at post transcriptional or transcriptional levels (Schauer et al., 2002). However, miRNAs, 20–24 nucleotides in length, are derived from endogenous primary transcripts upon processing. The processed mature miRNA is incorporated into the RISC complex and directs the RISC complex toward degradation or the translational inhibition of target mRNAs (Lee et al., 2004; Vazquez et al., 2010).

In plants, miRNAs are primarily associated with several physiological phenomena, such as growth, development, organogenesis, and responses to various biotic and abiotic stresses (Chen, 2004; Sunkar and Zhu, 2004; Kidner and Martienssen, 2005; Lu et al., 2008; Zhang et al., 2008; Kruszka et al., 2012). Recently, several novel miRNAs were reported for the uptake and transport of nutrient minerals in plants (Jones-Rhoades and Bartel, 2004; Fischer et al., 2013; Kehr, 2013). In the present review, current knowledge of the different classes of nutrient responsive-miRNAs and of their functions in nutrient homeostasis was extensively studied, which may aid in understanding the role of miRNAs as master regulators of nutrient loading in plants. The regulation of the differential expression of transporter genes by miRNAs may reveal a novel paradigm for crop biofortification or mineral bioavailability in cereal grains in the future.

## miRNAs in Nutrient Homeostasis

### miRNA and Phosphorus Nutrition

Inorganic phosphate (Pi), an essential mineral for plant growth and development is often a limiting factor in plant nutrition. Despite the adequate amount of phosphorus (P) in cultivated land, plants can uptake very small amounts of phosphorus due to its low availability. Plants synthesize several classes of nucleases and phosphatases to solubilize external Pi or to release Pi from organic substances and to upregulate the gene expression of certain exporter/importer transport proteins to acclimatize to phosphate starvation (Poirier and Bucher, 2002; Abel, 2011). Recent studies have identified the involvement of small RNAs, particularly miRNAs, in the differential regulation of phosphate-related gene expression in *Arabidopsis*, rice, wheat, barley, maize, soybean, white lupin, and tomato, etc. (**Table 1**; Pant et al., 2009; Gu et al., 2010; Lundmark et al., 2010; Zhu et al., 2010; Hackenberg et al., 2013; Pei et al., 2013; Xu et al., 2013; Zhao et al., 2013a). In *Arabidopsis*, the deprivation of Pi is rapidly transmitted to the shoots and induces the synthesis of miR399a-f families. miR399 binds to the five complementary sites of the phosphate over accumulator2 (*PHO2*) transcript, which is in the 5′ untranslated region (UTR), 200–400 bp upstream and induces mRNA cleavage (Pant et al., 2008). PHO2, an essential transporter for phosphate mobilization, is associated with E2 ubiquitin conjugating enzyme. The reduction of PHO2 helps to accumulate high amounts of Pi in the shoots. The role of miR399 and its biosynthesis regulation have been well demonstrated. The myeloblastosis (MYB) transcription factors like phosphate starvation response1 (PHR1) binding sites, such as *P1BS*, *PHO*, and *PHO*-like, are present upstream of the promoter region of miR399 (Bari et al., 2006; Zeng et al., 2010; Xu et al., 2013). The expression of miR399f is also regulated by the binding of MYB2 transcription factor at the *cis*-acting elements of the MIR399f promoter, as reported in *Arabidopsis* (Baek et al., 2013). Notably, long non-coding RNA, another signaling molecule, which is induced by phosphate starvation1 (IPS1), plays an inhibitory role by protecting the *PHO2* transcript and interferes with miR399-mediated *PHO2* gene regulation (Franco-Zorrilla et al., 2007; Hu et al., 2011; Huang et al., 2011).

**Table 1:** **Differentially expressed miRNAs reported under different nutrient/metal (N, P, S, Cu, Mn, Fe, Zn) deprivation in various plant species**.

micro RNA (miRNA)	Phosphorus (P)	Nitrogen (N)	Sulfur (S)	Copper (Cu)	Manganese (Mn)	Iron (Fe)	Zinc (Zn)
miR156	↑ (White lupin)	↑ (Arabidopsis)	↑ (Brassica napus)		↑ (Phaseolus vulgaris)		
miR156h		↑ (Arabidopsis)					
miR157					↑ (P. vulgaris)		
miR158	↑ (Tomato)					↑ (Arabidopsis)	
miR159	↑ (White lupin)					↑ (Arabidopsis)	
miR159a	↑ (Soybean)						
miR159b	↑ (Wheat)						
miR160	↑ (White lupin)	↑ (Arabidopsis, Maize)	↑ (B. napus)				
miR164	↑ (White lupin)	↑ (Maize leaves)	↑ (B. napus)		↑ (P. vulgaris)	↑ (Arabidopsis)	
miR166	↑ (White lupin)				↑ (P. vulgaris)		↑ (Sorghum bicolor)
miR167	↑ (Wheat)	↓ (Arabidopsis, ↑ maize)	↑ (B. napus)		↑ (P. vulgaris)		
miR168	↑ (White lupin)	↓ (maize roots)	↑ (B. napus)		↑ (P. vulgaris)		
miR169		(Arabidopsis, Medicago truncatula, maize)			↑ (P. vulgaris)		
miR169 (a-c)		↓ Arabidopsis					
miR169 (d-g)		↑ miR189g in Tomato)	↑ Soybean				
miR169 (h-n)	↓ Maize						
miR170	↓ Maize				↑ (P. vulgaris)		
miR171		↑ (Arabidopsis)					↑ (S. bicolor)
miR172	↑ (Tomato)	↑ (Arabidopsis, maize)			↑ (P. vulgaris)	↑ (Arabidopsis)	↑ (S. bicolor)
miR172b	↑ (Tomato)	↑ (Arabidopsis, maize)					
miR173	↑ (Tomato)					↑ (Arabidopsis)	
miR319	↑ (Tomato, White lupin)	↑ (Arabidopsis, maize)			↑ (P. vulgaris)		↑ (S. bicolor)
miR319a	↓ (Soybean)						
miR390	↓ (White lupin)				↑ (P. vulgaris)		
miR394			↑ (B. napus)			↑ (Arabidopsis)	
miR394			↑ (B. napus)			↑ (Arabidopsis)	
miR395	↑ (White lupin)	↓ ↑ (Arabidopsis, maize)	↑ (B. napus)		↑ (P. vulgaris)		
miR396a	↑ (White lupin)				↑ (P. vulgaris)		
	↓ (Soybean)				↑ (P. vulgaris)		
miR397		↑ (Maize)		↑ (Arabidopsis)		↓ (Arabidopsis)	
miR398a	↑ (Arabidopsis, Tomato)	↑ (Arabidopsis, maize)		↑ (Arabidopsis)		↓ (Arabidopsis)	↑ ↓ (S. bicolor)
miR398b	↓ (Soybean)					↓ (Arabidopsis)	
miR398c						↓ (Arabidopsis)	
miR398s						↓ (Arabidopsis)	
miR399(a-f)	↑ (Arabidopsis, Wheat, Tomato)	↓ ↑ (Arabidopsis, maize)	↑ (B. napus)			↓ (Arabidopsis)	↑ (S. bicolor)
miR408	↓ (Wheat)	↑ (Maize)		↑ (Arabidopsis)		↓ (Arabidopsis)	
miR437	↑ (White lupin)						
miR447	↓ (White lupin)						
miR528		↑ (Maize)					↓ (S. bicolor)
miR771	↓ (Tomato)						
miR775	↓ (Tomato)						
miR778	↑ (Arabidopsis)						
miR826		↓ (Arabidopsis)					
miR827	↑ (Arabidopsis)	↓ (Rice, maize)					
miR829		↑ (Arabidopsis)					
miR830	↑ (White lupin)						
miR837-3p	↑ (Tomato)						
miR839		↑ (Arabidopsis)					
miR846		↑ (Arabidopsis)					
miR850		↓ (Arabidopsis)					
miR857	↑ (White lupin)	↓ (Arabidopsis)		↑ (Arabidopsis)			
miR863		↓ (Arabidopsis)					
miR896	↑ (White lupin)						
miR1122	↑ (Wheat)						
miR1125	↑ (Wheat)						
miR1135	↑ (Wheat)						
miR1136	↑ (Wheat)						
miR1211	↓ (White lupin)						
miR1222	↑ (White Lupin)						
miR1507a	↓ (Soybean)						
miR2111	↑ (Arabidopsis)	↓ (Arabidopsis)		↑ (Arabidopsis)		↓ (Arabidopsis)	

In *Arabidopsis,* phosphate limitation also increases the amounts of three other miRNAs: miR778, miR827, and miR2111. Intriguingly, within 3 h of Pi re-addition, the abundance of miR2111, and miR778 rapidly reduce by approximately twofold. Two copies of miR2111 are present in the *Arabidopsis* genome, and both loci show similar phosphate response activities (Pant et al., 2009). Recently, the dual responses of miR827, miR399, miR2111, and miR827 have been elucidated. The activity of miR2111 is reversed under nitrogen (N)-starvation compared to phosphate (P)-starvation (Liang et al., 2012). The miR827 and miR399 have also been identified to function in N-starvation by targeting the *nitrogen limitation adaptation* (*NLA*) gene and are thought to increase the expression of the PHO2 transporter. In lower abundances of nitrates, the *nla* mutant has been found to play an important role in phosphorus homeostasis by accumulating excessive Pi. A similar condition has been observed for miR827-overexpressing *Arabidopsis* plants and supports the miR827-mediated gene regulation of phosphate transporters (Kant et al., 2011). In rice, Pi accumulation followed by a perturbation of phosphorus mobilization in old leaves is also manifested by miR827 (Wang et al., 2012). Notably, the expression of miR398a is regulated by P, carbon (C) and N limitation, indicating a more general role in mineral homeostasis. Repression due to C limitation was also shown to be correlated with the induction by sucrose (Dugas and Bartel, 2008). Furthermore, soybean roots, miR159a has been found to be up regulated during P deficiency, whereas down regulation of miR319a, miR396a, miR398b, miR1507a has been shown. The differential regulation of miRNAs during P-starvation depends on the frequency of phosphorus responsive motifs (P responsive motifs) in the promoter of the miRNA genes. The number of P-responsive motifs in the *cis*-acting region of miRNA genes is reported to be higher than those of non-responding miRNA genes (Zeng et al., 2010).

### miRNAs in Nitrogen Uptake and Transport

Nitrogen, an essential constituent for nucleic acids, protein, chlorophyll, etc., plays a crucial role in plant growth and development. N transport depends on the external acquisition of N by roots from the soil, except the mechanism of biological N fixation in legumes. Plants can adapt in N-limiting soil conditions by up- or down-regulating a specific group of exporter or importer proteins. The differential regulation of transporters is selectively controlled by several small miRNA families. In *Arabidopsis*, depending on the occurrence at N-starvation, the miRNA-responsive populations can be categorized into two groups by Solexa high-throughput sequencing. N-starvation-induced (NSI) miRNA families include several members, such as miR156, miR169, miR171, miR160, miR319, miR826, miR829, miR839, and miR846, whereas miR167, miR172, miR399, miR395, miR850, miR857, miR863, and miR827 are recognized as N-starvation-suppressed (NSS) miRNA group members (Liang et al., 2012; **Table 1**). Furthermore, 15 and 14 miRNA families have been identified to be responsive in N-limiting conditions in rice and maize, respectively (Xu et al., 2011; Nischal et al., 2012). The miR156 family in *Arabidopsis* has been found at the highest abundance, and miR156h is thought to be the most important among the three members of the miR156 family. On the contrary, miR172 is negatively regulated by miR156 and inhibits the reproductive phase by prolonging the juvenile period. In N-starvation, the induction of miRNA160 inhibits lateral root development, whereas miR170 hastens the growth of primary roots by targeting auxin response factor (ARF16/17) and SCL6 regulatory proteins, respectively (Liang et al., 2012). In contrast, the perturbation of miR167 biogenesis in N-limiting condition attenuates the expression of ARF6/8, which in turn facilitates the development of lateral and adventitious roots (Jones-Rhoades and Bartel, 2004; Gifford et al., 2008).

In *Medicago truncatula*, miR169 and miR172 play a pivotal role in nodule development by regulating the expression of the *HAP2* and *AP2* genes. The lower abundances of miR169 during N-limitation upregulates *HAP2* gene expression and the subsequent differentiation of nodule primordial by maintaining low N in the roots (Pant et al., 2009). However, different members of the miR169 family, such as miR169a, miR169bc, miR169d-g, and miR169h-n, have been reported in different plant species of *Arabidopsis*, soybean, and maize, etc. They are actively associated with the up-regulation of nitrate transporters during N-starvation (Xu et al., 2011; Zhao et al., 2011, 2012, 2013b; Liang et al., 2012; Wang et al., 2013b). Under low N availability, the expression of miR169d-g has been shown to be increased. miR169d-g exhibits a similar pattern of expression during P and sulfur (S) deficiencies, whereas other members are expressed differently (Liang et al., 2012). Very recently, miR172 was found to be exclusively expressed in the nodules of soybeans, targeting *Arabidopsis* homologous gene *APETALA*2-related transcription factors in response to P-starvation (Yan et al., 2013).

### miRNAs in Sulfur Homeostasis

Sulfur (S), an indispensable inorganic mineral, is mainly taken up by roots in the form of sulfate from the soil. The S is assimilated into cysteine, methionine, glutathione, glucosinolate compounds, and various Fe–S proteins, cofactors, and lipoic acids, which are associated with both primary and secondary metabolism during stress (Rausch and Wachter, 2005). In *Arabidopsis*, sulfate is transported through xylem or phloem via cell-specific transporters such as sulfate transporters1;1 (SULTR1;1), SULTR2;1, and SULTR2;2. The expression of the transporters is predominantly regulated by miR395 depending on S starvation or abundance (Kawashima et al., 2009). Intriguingly, sulfate limitation induces the expression of miR395 and its low affinity sulfate transporter SULTR2;1, in contrast to the inhibitory effect of sulfate deficiency. SULTR2;1 is primarily confined to the xylem parenchyma, whereas miR395 is highly abundant in the phloem parenchyma and plays crucial role in sulfate remobilization between leaves during sulfate deficiency (Liang and Yu, 2010). The restriction of SULTR2;1 expression by miR395 in the xylem parenchyma facilitates the translocation of sulfate ions from the roots to the shoots. In addition, S deficiency leads to the elevated synthesis of SULFUR LIMITATION1 (SLIM1) protein in the roots, which in turn activates various sulfate transporters to enhance S uptake (Liang et al., 2010; Kawashima et al., 2011). The role of miR395 has also been elucidated in S assimilation by suppressing the expression of ATP sulfurylase genes, such as *APS1*, *APS3*, and *APS4*, which catalyze the first step of S assimilation (Matthewman et al., 2012). The expression levels of miR156, miR160, miR164, miR167, miR168, and miR394 are also modulated by S deprivation, as observed in *Brassica* *napus* (Huang et al., 2010).

### miRNAs in Copper Homeostasis

Copper (Cu) is an essential micronutrient that serves primarily as a cofactor of metabolic enzymes and protein complexes in the electron transport chain. It is an integral member of plastocyanin, which actively participates in the electron transport of chloroplast grana during photosynthesis. Cu plays an important role against oxidative stress responses by acting as a cofactor of Copper/Zinc superoxide dismutase (CSD). During Cu limitation, the induction of miR398 down regulates *CSD1*, *CSD2,* and Cu chaperones for superoxide dismutase *SOD1* (*CCS1*) gene expression (Sunkar et al., 2006; Beauclair et al., 2010). CCS1 is a chaperone protein that delivers the Cu ions to *CSD1* and *CSD2* apoprotein. Under Cu-deficient conditions, Cu/Zn superoxide function is replaced by iron (Fe) superoxide dismutase due to the low availability of Cu. The subunit of cytochrome C oxidase, the inner membrane protein of mitochondria encoded by *CYCLO MONOOXYGENASE* is also repressed by miR398 expression in *Arabidopsis* (Abdel-Ghany and Pilon, 2008). Other families of miRNAs, such as miR397, miR408, and miR857, have been found to be up regulated during Cu starvation, which in turn suppresses the expression of laccase and plastocyanin genes. The three laccase genes (*LAC*), such as *LAC3*, *LAC12*, and *LAC13*, are down regulated by miR408, and the mRNA of *LAC2*, *LAC4,* and *LAC17* are degraded by miR397. miR857 is primarily responsible for targeting *LAC7* transcripts. Notably, miRNAs related to Cu homeostasis facilitate plastocyanin biosynthesis by reducing the biosynthesis of non-essential Cu enzymes, thus ensuring Cu homeostasis by altering Cu availability among various groups of proteins (Gifford et al., 2008).

### miRNA in Other Mineral Homeostasis

Manganese (Mn), Fe, and Zinc (Zn) are essential minerals for plant growth and nutrition. Several miRNAs have been found to be up regulated during Mn starvation and are also associated with other mineral stresses. In *Phaseolus vulgaris*, miR319, miR169, miR396, miR170, miR164, miR390, miR395, miR166, miR172, miR157, miR156, and miR167 are up regulated during Mn toxicity and attenuate the expression of a wide group of genes, including various transcription factors such as *TEOSINTE-LIKE1, CYCLOIDEA*, *PROLIFERATING CELL FACTOR1 (TCP*), *HAPLESS (HAP2*), *SCARECROW-LIKE, NO APICAL MERISTEM* (*NAC), Arabidopsis* transcription activation factor, *CUP SHAPED COTYLEDON,* serine threonine protein kinase, and *APETALA2*, etc. (Valdes-Lopez et al., 2010).

Iron and Zn are indispensable micronutrients for plants, as they are the major cofactors for several key metabolic enzymes, including Fe-S cluster proteins and ferredoxin molecules (Couturier et al., 2013; Forieri et al., 2013). Furthermore, the inadequate amounts of Fe and Zn that are stored in the edible parts of cereal grains play immense roles in human nutrition. The bioavailability of minerals in grains is directly associated with the uptake, transport, and loading of mineral ions (Aung et al., 2013). Recently, the miRNA-mediated regulation of Fe-related transporters and storage proteins were elucidated in *Arabidopsis*. miR398, one of the key activators for *CSD* gene expression during Cu deficiency, is also regulated by Fe deficiency but in an opposite manner. Fe deficiency reduces the expression of miR397, miR398a, miR398b, miR398c, miR398s, miR399, miR408, and miR2111, whereas Cu deficiency increases their expression and in turn regulates the expression of *CSD1* and *CSD2* (Buhtz et al., 2010; Waters et al., 2012). Therefore, the Cu-Fe interrelationship is another novel finding regarding the study of gene expression during Fe homeostasis. In addition, eight miRNAs from five families, including miR159, miR164, miR172, miR173, and miR394, were previously identified as Fe-responsive families from the small RNA library population in *Arabidopsis.* Intriguingly, the Fe deficiency responsive *cis*-acting elements1 and 2 (*IDE1/IDE2*) were found within the promoters of twenty-four miRNA genes in *Arabidopsis* and resemble the Fe-responsive gene families that are regulated during Fe deficiencies (Kong and Yang, 2010). However, the roles of other miRNAs in Fe transport and storage have not been clearly established.

The Zn deficiency in *Sorghum bicolor* aggravates the upregulation of several miRNA families, such as miR166, miR171, miR172, miR398, miR399, and miR319, which in turn target many gene family members including transporters (Li et al., 2013). Interestingly, two miRNA family members were found to be involved in the regulation of the expression of the *CSD* gene family but in an opposite manner. The upregulation of miR398 reduces the gene expression of *CSD* in the roots, whereas attenuated miR528 elevate the level of *CSD* transcripts in the seeds and the leaves. Furthermore, miRNAs maintain nutrient homeostasis by possessing an endogenous signal for the transport of micro and macronutrients (Liu et al., 2009; Marín-González and Suárez-López, 2012; Kehr, 2013). The up- or downregulation of diverse miRNAs during other metal stresses, such as Al, Cd, Hg, and Cd stress, have also been reported and likely play a role in the adaptive mechanisms of plants by regulating the expression of various stress-related genes (Zeng et al., 2012; Zhou et al., 2012; Zhang et al., 2013).

## miRNAs in Systemic Mobility and Long-Distance Transport

After uptake by the roots, nutrients are allocated to the various storage parts of plants following long-distance transport. Xylem and phloem contribute to long-distance transport, and phloem-mediated communication plays an important role during nutrient stress. Phloem not only preserves the source-sink relationship but it also ensures cell-to-cell signal communication during different biotic and abiotic stress responses. Increasing evidence suggests that phloem-specific mRNAs coupled with small RNAs act as signaling molecules in different physiological responses, including nutrient transport under low nutrient conditions in several plant species (Varkonyi-Gasic et al., 2010; Kehr, 2013). Recently, microarrays of *B. napus* have revealed the presence of a specific set of phloem sap-specific miRNAs that are accumulated during S and Cu deficiency and are distinct in the roots, leaves, and inflorescence axis (Buhtz et al., 2010). miR395, which is known as sulfur deficiency responsive-miRNA, accumulates in phloem sap with miR399 and miR2111. miR399 and miR2111, the phosphate starvation responsive miRNAs, have also been found in phloem sap under Cu-deficient conditions (Abdel-Ghany and Pilon, 2008; Pant et al., 2008; Buhtz et al., 2010). miR399d, the member of miRNA399 family, exhibits long-distance transport from the shoots to the roots via phloem, conjugated with small RNA binding proteins, exemplified by *Cucurbita maxima* phloem small RNA binding protein 1 (*Cm*PSRP1) and *C. maxima* phloem protein 16 (*Cm*PP16; Pant et al., 2009). Furthermore, grafting experiments in the *Arabidopsis* mutant, *hen-1-1*, corroborates the mobility of miR399 and miR395 from the shoots to the roots via phloem, thus transmitting signals during nutrient deficiency. Interestingly, the translocation of miR395 was found to down-regulate only *APS4* but not *APS1* or *AtSULTR2;1*. On the other hand, miR158 in phloem sap appears to play an important role in nutrient transport by targeting lipase and xyloglucan fucosyltransferase genes during Fe deficiency, whereas miR172 was found to play an essential role in tuber formation and is considered as a phloem-specific signaling intermediate for plant growth and development (Martin et al., 2009; Kasai et al., 2010).

## Conserved Groups of miRNAs in Nutrient Homeostasis

microRNAs play an crucial role in nutrient homeostasis by altering gene expression in plants. A single miRNA family has been reported to take part in different nutrient homeostasis conditions, thus playing orchestrated roles as signaling intermediates in several metabolic pathways. For example, two families of miRNAs, the miR169 and miR172 families are exclusively found in nodules and are involved in N, P, and Mn stresses. In addition, elevated levels of miR167 and miR395 during N and S starvation have been reported. These findings can be attributed to the fact that some common transcription factors activated by N and S-stress responses are responsible for the biosynthesis of these two miRNAs. The up-regulation of miR319 and miR396 during N and Mn starvation also supports the hypothesis (Valdes-Lopez et al., 2010). In this review, based on the abundances during various nutritional stresses, many families of miRNAs can be categorized into four conserved groups (**Table 2**). In different plant species, miR164, miR172, miR398, and miR399 are involved in the homeostasis of five different nutrients, thus representing the highest conserved group. miR156, miR167, miR395, miR319, miR408, and miR2111 are classified as highly conserved depending on their up-regulation during the four types of nutrient stress responses. miR160, miR168, miR166, miR397, and miR857 are categorized as moderately conserved (abundance frequencies three times), and miR158, miR159, miR169, miR170, miR171, miR528, miR390, miR396, miR394, and miR827 belong to the least conserved group (twice abundance frequencies). A particular miRNA can regulate different nutritional homeostasis conditions by up- or down-regulating the expression of various target genes (**Table 2**), which suggests a common signaling role of m), which suggests a common signaling role of miRNAs in the regulation of diverse nutritional stress responses. Other unique miRNAs also have been identified in some plant species during particular nutrient stress responses, and their roles in the homeostasis of other nutrients should be investigated in the future.

**Table 2:** **Categorization of “conserved” and “unique” miRNAs under different nutrient stress and their predicted target genes**.

Nature of miRNA families		miR ID	Frequencies of occurrence under different low/high nutrient conditions	Predicted target genes
Conserved	Highest	miR164	5 (P^1^, N^2^, S^3^, Mn^5^, Fe^6^)	*PHO* 2 ^1^, *NLA*^2^, *AP*2^1,2,3^, APS1^3^, *CSD*1^4,6^, *CSD*2^4,6^, *SOD*1^4^, *CYCLOIDEA*^5^, Fe–S Cluster Proteins^6^, Fe transporters^6,7^
		miR172	5 (P^1^, N^2^, Mn^5^, Fe^6^, Zn^7^)	
		miR398	5 (P^1^, N^2^, Cu^4^, Fe^6^, Zn^7^)	
		miR399	5 (P^1^, N^2^, S^3^, Fe^6^, Zn^7^)	
	High	miR156, miR167, miR395	4 (P^1^, N^2^, S^3^, Mn^5^)	*ARF* 6 ^1^, *ARF* 8^1,2^, *NAC* ^5^, *TEOSINTE-LIKE* 1^5^ *SCARECROW-LIKE* ^5^, *APS*1^3^, *APS* 3^3^, *APS* 4^3^, *SULTR* 1;1^3^, *SULTR* 2;1^3^, *SULTR* 2;2^3^, *CSD* 1^4^, *CSD* 2^4^, Fe transporters^6,7^
		miR319	4 (P^1^, N^2^, Mn^5^, Zn^7^)	
		miR408, miR2111	4 (P^1^, N^2^, Cu^4^, Fe^6^)	
	Moderate	miR160, miR168	3 (P^1^, N^2^, S^3^)	*ARF* 16^2^, *ARF* 17^1,2^, *NLA* ^2^, *APS* 1^3^, *LAC* 2^4^,*LAC* 4^4^, *LAC* 7^4^, *LAC* 17^4^, *PLASTOCYNIN*^4^, Fe–S Cluster ^Proteins3,6^, Fe transporters^6,7^
		miR166	3 (P^1^, N^2^, Zn^7^)	
		miR397	3 (N^2^, Cu^4^, Fe^6^)	
		miR857	3 (P^1^, N^2^, Cu^4^)	
	Less	miR158, miR159	2 (P^1^, Fe^6^)	*HAP* 2^2^, *PROLIFERATING CELL FACTOR* 1^5^, *SCL* 6^2^, *NLA* ^2^, *SERINE THREONINE PROTEIN KINASE* ^5^, *AP* 2^1,2,3^, Fe-S Cluster Proteins^3,6^, *FERRIDOXIN* ^6^, Iron transporters^6,7^
		miR169, miR170	2 (N^2^, Mn^5^)	
		miR171, miR528	2 (N^2^, Zn^7^)	
		miR390, miR396	2 (P^1^, Mn^5^)	
		miR394	2 (S^3^, Fe^6^)	
		miR827	2 (P^1^, N^2^)	
Unique		miR437, miR447, miR771, miR775, miR778, miR830, miR837, miR896, miR1122, miR1125, miR1135, miR1136, miR1211, miR1222, miR1507	P	*PHO2, ARF*6, ARF8, AP2
		miR826, miR829, miR839, miR846, miR850, miR863,	N	*NLA, ARF*6 *,ARF*8 *,ARF*16*, ARF*18, *HAP2, AP*2
		miR173	Fe	Fe–S Cluster Proteins, *FERRIDOXIN*, Fe transporters

*1,2,3,4,5,6,7 denote P, N, S, Cu, Mn, Fe, and Zn-mediated target gene regulation, respectively.*

## Concluding Remarks and Future Perspectives

Major research endeavors have focused on the genetic regulation of P and N transporters under the respective nutrient stress conditions. Future approaches for miRNA-mediated regulation of nutrient transporters and other metabolic enzymes and their implementation in future biotechnological research are summarized in **Table 3**. Because phytate is an important source of inorganic phosphorus, the role of miRNAs in phosphate metabolism, including inositol phosphate or phytic acid biosynthesis is a promising arena for future research. Nitrate metabolism and the N to C ratio determine the biomass of cereals. The roles of miRNA in the regulation of nitrate transporters and metabolism, including several enzymes such as aspartate amino transferase, glutamine synthase, and glutamate dehydrogenase, should be more extensively investigated. Regarding metal homeostasis, future investigations on differentially expressed-miRNAs and their regulatory roles in various Fe and Zn transporters may aid in the development of a novel platform for Fe and Zn loading in cereal grains. Fe and Zn, two important dietary nutrients, are found only in small amount in the consumable parts of cereal grains. To increase the content of these metals in milled grain, various biotechnological strategies have been utilized (Paul et al., 2013; Wang et al., 2013a; Borrill et al., 2014; Khan et al., 2014). However, the role of miRNAs in regulating specific transporters or transcription factors in Fe nutrition has not been studied extensively to date. The Fe-related gene regulation mechanism is important for understanding Fe nutrition and may elucidate the clear scenario of gene regulation during nutrient homeostasis.

**Table 3:** **Some probable future strategies for improvement of plant nutrition associated with miRNAs research**.

Future strategies	Purpose to be solved
Investigation of novel miRNAs and their role in phytate biosynthesis like regulation of different inositol phosphate kinase genes, alteration of specific miRNA expression by overexpression or genome editing	Alternative approach for combating the phytate barrier in grains to increase the mineral biavailability
Role of miRNAs in regulation of nitrate transporters and metabolic enzymes such as aspartate amino transferase, glutamine synthase, glutamate dehydrogenase	Improvement of biomass production in crops (since nitrogen and carbon ration is crucial for biomass)
Role of miRNAs in different groups of Fe and Zn transporters from roots to seed, miRNA promoter/ genome editing	Improvement of transport and allocation of Fe and Zn in seeds
Identification of root specific novel miRNAs under nutrient stress and investigation the role of miRNA- mediated miRNA activation or removal of supressors of transporters	Improvement of nutrient uptake by roots by overexpression of miRNAs
Novel phloem-specific miRNAs under nutrient stress	Studying the signal transduction mechanism during long distance transport, interconnecting relationships among different nutrient transport
Novel miRNA under different nutrient stress	Central signaling role of regulatory network between different metabolic pathways
Role of miRNAs in down-regulation of heavy metal transporters	Development of heavy metals or arsenic tolerant plants by overexpressing specific group of miRNAs

**FIGURE 1:**
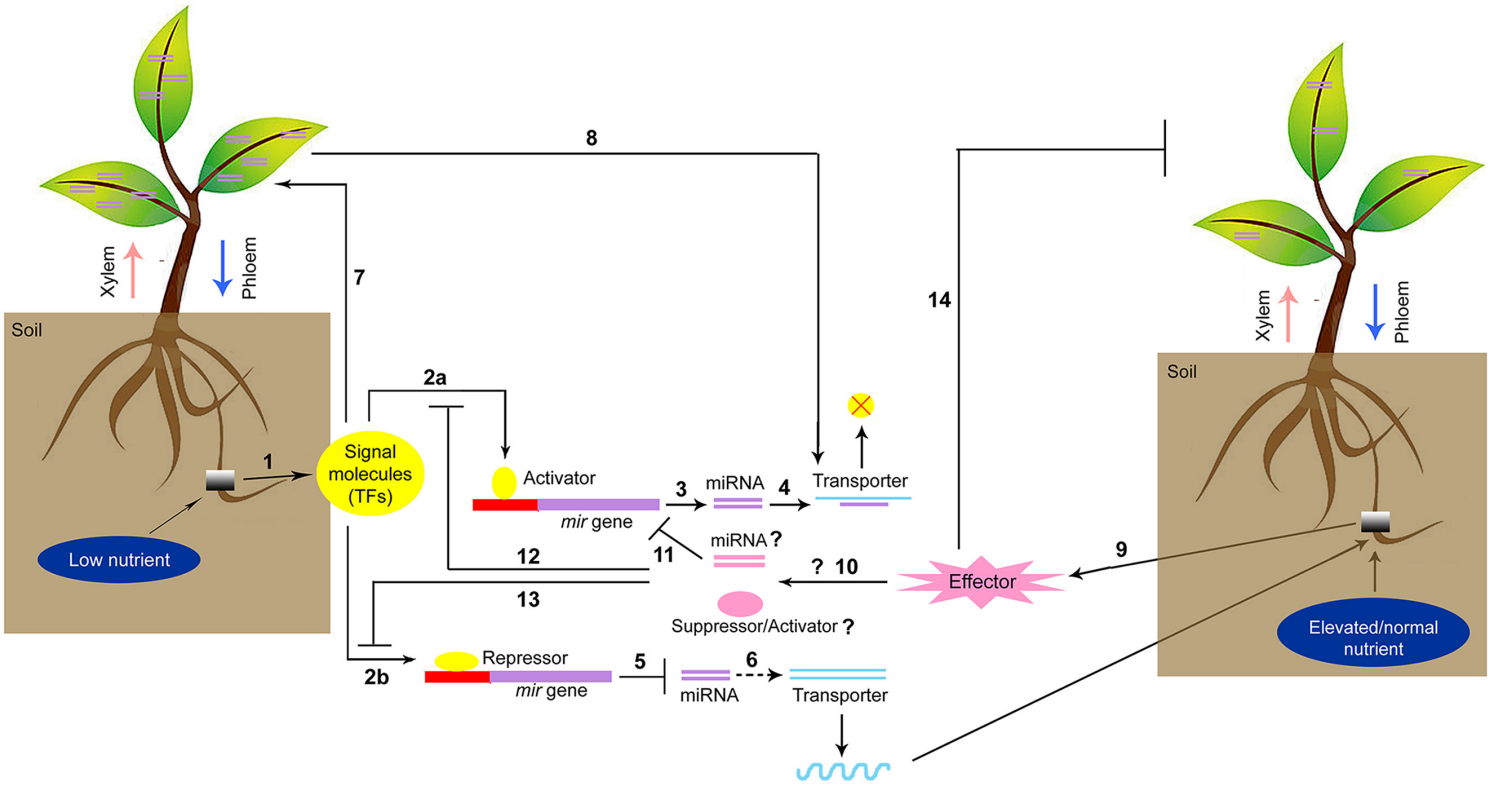
**Proposed model of miRNA-mediated signaling network for regulation of nutrient homeostasis**. (1) Low nutrient in soil upregulates signaling molecules which can act as an activator of nutrient stress responsive miRNA biosynthesis following interaction with *mir* promoter (2a) or repress the miRNA biosynthesis (2b) Activation of miRNA (3) attenuates the transporter gene expression (4) Reduction of miRNA biosynthesis (5) further stabilizes transporter gene expression (6) and more nutrients are transported. Signal molecules can induce phloem specific small or miRNA biosynthesis (7) which in turn regulates the mobility of nutrients through phloem by activating the transporters (8) Elevated or ambient condition of nutrients can induce other signal molecules which act as an effector (9) and facilitate the biosynthesis of other groups of miRNA or small RNAs (10) that interact with low nutrient stress responsive-miRNA in a opposite way (11) stimulating the specific group of transporters. The effector molecules may act as suppressor against the low nutrient stress responsive-activator (12) or inducer to mitigate the activity of low nutrient stress responsive-activator (13) Effector molecules can also reduce the biosynthesis of phloem specific miRNA or small RNA families and the phloem mediated transport is regulated.

microRNAs-mediated signal transduction during low/high nutrient stress is a fascinating topic of plant nutrition research. The alteration of nutrient levels in soil can trigger specific signaling molecules that act as repressors of target nutrient responsive-miRNAs. The decreased accumulation of miRNAs subsequently stabilizes the expression of transporters (**Figure 1**). On contrary, the optimal conditions or higher amounts of nutrients can trigger a specific group of miRNAs/small RNAs that directly affect the transporter (as exemplified by the phosphate transporter) or induce other miRNAs that suppress the expression of repressor genes. Therefore, the differential expression of miRNAs and their regulation under nutrient stress provide valuable information. The discovery of phloem-specific novel miRNAs during nutrient starvation and their cell-to-cell transmission will lead to a better understanding of the interrelationship among different nutrients. The identification of promoter regions of specific up- or down-regulated miRNAs that are responsive to micronutrient stresses and the subsequent development of knock-out mutants by inducing mutation in *cis*-acting elements using targeted genome-editing technologies, such as transcription activator-like effector nuclease (TALEN) or clustered regularly interspaced short palindromic repeats -CRISPR-associated 9 (CRISPR-Cas9) techniques, may lead to essential crop-improvement strategies in the future.

## Conflict of Interest Statement

The authors declare that the research was conducted in the absence of any commercial or financial relationships that could be construed as a potential conflict of interest.

## References

[B1] Abdel-Ghany S. E. Pilon M. (2008). MicroRNA-mediated systemic down-regulation of copper protein expression in response to low copper availability in *Arabidopsis*. *J. Biol. Chem.* 283 15932–15945 10.1074/jbc.M801406200 18408011 PMC3259626

[B2] Abel S. (2011). Phosphate sensing in root development. *Curr. Opin. Plant Biol.* 14 303–309 10.1016/j.pbi.2011.04.007 21571579

[B3] Aung M. S. Masuda H. Kobayashi T. Nakanishi H. Yamakawa T. Nishizawa N. K. (2013). Iron biofortification of Myanmar rice. *Front. Plant Sci.* 4:158 10.3389/fpls.2013.00158 PMC3664312 23750162

[B4] Baek D. Kim M. C. Chun H. J. Kang S. Park H. C. Shin G. (2013). Regulation of miR399f transcription by AtMYB2 affects phosphate starvation responses in *Arabidopsis*. *Plant Physiol.* 161 362–373 10.1104/pp.112.205922 23154535 PMC3532267

[B5] Bari R. Pant B. D. Stitt M. Scheible W. R. (2006). PHO2 microRNA399 and PHR1 define a phosphate-signaling pathway in plants. *Plant Physiol.* 141 988–999 10.1104/pp.106.079707 16679424 PMC1489890

[B6] Bartel D. P. (2004). MicroRNAs: genomics, biogenesis, mechanism, and function. *Cell* 116 281–297 10.1016/S0092-8674(04)00045-5 14744438

[B7] Beauclair L. Yu A. Bouche N. (2010). microRNA directed cleavage and translational repression of the copper chaperone for superoxide dismutase mRNA in *Arabidopsis. Plant J* 62 454–462 10.1111/j.1365-313X.2010.04162.x 20128885

[B8] Borrill P. Connorton J. M. Balk J. Miller A. J. Sanders D. Uauy C. (2014). Biofortification of wheat grain with iron and zinc: integrating novel genomic resources and knowledge from model crops. *Front. Plant Sci.* 5:53 10.3389/fpls.2014.00053 PMC3930855 24600464

[B9] Buhtz A. Pieritz J. Springer F. Kehr J. (2010). Phloem small RNAs, nutrient stress responses, and systemic mobility. *BMC Plant Biol.* 10:64 10.1186/1471-2229-10-64 PMC2923538 20388194

[B10] Chen X. (2004). A microRNA as a translational repressor of APETALA2 in *Arabidopsis* flower development. *Science* 303 2022–2025 10.1126/science.1088060 12893888 PMC5127708

[B11] Couturier J. Touraine B. Briat J.-F. Gaymard F. Rouhier N. (2013). The iron-sulfur cluster assembly machineries in plants: current knowledge and open questions. *Front. Plant Sci.* 4:259 10.3389/fpls.2013.00259 PMC3721309 23898337

[B12] Dugas D. V. Bartel B. (2008). Sucrose induction of *Arabidopsis* miR398 represses two Cu/Zn superoxide dismutases. *Plant Mol. Biol.* 67 403–417 10.1007/s11103-008-9329-1 18392778

[B13] Fischer J. J. Beatty P. H. Good A. G. Muench D. G. (2013). Manipulation of microRNA expression to improve nitrogen use efficiency. *Plant Sci.* 210 70–81 10.1016/j.plantsci.2013.05.009 23849115

[B14] Forieri I. Wirtz M. Hell R. (2013). Toward new perspectives on the interaction of iron and sulfur metabolism in plants. *Front. Plant Sci.* 4:357 10.3389/fpls.2013.00357 PMC3788360 24106494

[B15] Franco-Zorrilla J. M. Valli A. Todesco M. Mateos I. Puga M. I. Rubio-Somoza I. (2007). Target mimicry provides a new mechanism for regulation of microRNA activity. *Nat. Genet.* 39 1033–1037 10.1038/ng2079 17643101

[B16] Gifford M. L. Dean A. Gutierrez R. A. Coruzzi G. M. Birnbaum K. D. (2008). Cell-specific nitrogen responses mediate developmental plasticity. *Proc. Natl. Acad. Sci. U.S.A.* 105 803–808 10.1073/pnas.0709559105 18180456 PMC2206617

[B17] Gu M. Xu K. Chen A. Zhu Y. Tang G. Xu G. (2010). Expression analysis suggests potential roles of microRNAs for phosphate and arbuscular mycorrhizal signaling in *Solanum lycopersicum*. *Physiol. Plant.* 138 226–237 10.1111/j.1399-3054.2009.01320 20015123

[B18] Hackenberg M. Huang P. J. Huang C. Y. Shi B. J. Gustafson P. Langridge P. A. (2013). Comprehensive expression profile of microRNAs and other classes of non-coding small RNAs in barley under phosphorous-deficient and -sufficient conditions. *DNA Res.* 20 109–125 10.1093/dnares/dss037 23266877 PMC3628442

[B19] Hu B. Zhu C. Li F. Tang J. Wang Y. Lin A. (2011). LEAF TIP NECROSIS1 plays a pivotal role in the regulation of multiple phosphate starvation responses in rice. *Plant Physiol.* 156 1101–1115 10.1104/pp.110.170209 21317339 PMC3135962

[B20] Huang C. Y. Shirley N. Genc Y. Shi B. Langridge P. (2011). Phosphate utilization efficiency correlates with expression of low-affinity phosphate transporters and noncoding RNA, IPS1 in barley. *Plant Physiol.* 156 1217–1229 10.1104/pp.111.178459 21606317 PMC3135919

[B21] Huang S. Q. Xiang A. L. Che L. L. Chen S. Li H. Song J. B. (2010). A set of miRNAs *Brassica napus* in response to sulphate deficiency and cadmium stress. *Plant Biotechol. J.* 8 887–899 10.1111/j.1467-7652.2010.00517 20444207

[B22] Jamalkandi S. A. Masoudi-Nejad A. (2009). Reconstruction of *Arabidopsis thaliana* fully integrated small RNA pathway. *Funct. Integr. Genomics* 9 419–432 10.1007/s10142-009-0141-z 19802639

[B23] Jones-Rhoades M. W. Bartel D. P. (2004). Computational identification of plant microRNAs and their targets, including a stress-induced miRNA. *Mol. Cell.* 14 787–99 10.1016/j.molcel.2004.05.027 15200956

[B24] Jung J. K. H. McCouch S. (2013). Getting to the roots of it: genetic and hormonal control of root architecture. *Front. Plant Sci.* 4:186 10.3389/fpls.2013.00186 PMC3685011 23785372

[B25] Kant S. Peng M. Rothstein S. J. (2011). Genetic regulation by NLA and microRNA827 for maintaining nitrate-dependent phosphate homeostasis in *Arabidopsis*. *PLoS Genet.* 7:e1002021 10.1371/journal.pgen.1002021 PMC3063762 21455488

[B26] Kasai A. Kanehira A. Harada T. (2010). miR172 can move longdistances in *Nicotiana benthamiana*. *Open Plant Sci. J.* 4 1–6.

[B27] Kawashima C. G. Matthewman C. A. Huang S. Lee B. R. Yoshimoto N. Koprivova A. (2011). Interplay of SLIM1 and miR395 in the regulation of sulfate assimilation *Arabidopsis*. *Plant J.* 66 863–876 10.1111/j.1365-313X.2011.04547.x 21401744

[B28] Kawashima C. G. Yoshimoto N. Maruyama-Nakashita A. Tsuchiya Y. N. Saito K. Takahashi H. (2009). Sulphur starvation induces the expression of microRNA-395 and one of its target genes but in different cell types. *Plant J.* 57 313–321 10.1111/j.1365-313X.2008.03690.x 18801012

[B29] Kehr J. (2013). Systemic regulation of mineral homeostasis by micro RNAs. *Front. Plant Sci.* 4:145 10.3389/fpls.2013.00145 PMC3655319 23720667

[B30] Khan M. A. Castro-Guerrero N. Mendoza-Cozatl D. G. (2014). Moving toward a precise nutrition: preferential loading of seeds with essential nutrients over non-essential toxic elements. *Front. Plant Sci.* 5:51 10.3389/fpls.2014.00051 PMC3929903 24600463

[B31] Khraiwesh B. Zhu J. K. Zhu J. (2012). Role of miRNAs and siRNAs in biotic and abiotic stress responses of plants. *Biochim. Biophys. Acta* 1819 137–148 10.1016/j.bbagrm.2011.05.001 21605713 PMC3175014

[B32] Kidner C. A. Martienssen R. A. (2005). The developmental role of microRNA in plants. *Curr. Opin. Plant Biol.* 8 38–44 10.1016/j.pbi.2004.11.008 15653398

[B33] Kong W. W. Yang Z. M. (2010). Identification of iron-deficiency responsive microRNA genes and cis-elements in *Arabidopsis*. *Plant Physiol. Biochem.* 48 153–159 10.1016/j.plaphy.2009.12.008 20097571

[B34] Kruszka K. Pieczynski M. Windels D. Bielewicz D. Jarmolowski A. Kulinska-Szweykowska Z. (2012). Role of micro RNAs and other sRNAs of plants in their changing environments. *J. Plant Physiol.* 169 1664–1672 10.1016/j.jplph.2012.03.009 22647959

[B35] Lee Y. Kim M. Han J. Yeom K. H. Lee S. Baek S. H. (2004). MicroRNA genes are transcribed by polymerase II. *EMBO J.* 23 4051–4060 10.1038/sj.emboj.7600385 15372072 PMC524334

[B36] Li Y. Zhang Y. Shi D. Liu X. Qin J. Ge Q. (2013). Spatial-temporal analysis of zinc homeostasis reveals the response mechanisms to acute zinc deficiency in *Sorghum bicolor*. *New Phytol.* 200 1102–1115 10.1111/nph.12434 23915383

[B37] Liang G. He H. Yu D. (2012). Identification of nitrogen starvation-responsive miRNAs in *Arabidopsis thaliana*. *PLoS ONE* 7:e48951 10.1371/journal.pone.0048951 PMC3498362 23155433

[B38] Liang G. Yang F. Yu D. (2010). MicroRNA395 mediates regulation of sulfate accumulation and allocation in *Arabidopsis thaliana*. *Plant J.* 62 1046–1057 10.1111/j.1365-313X.2010.04216.x 20374528

[B39] Liang G. Yu D. (2010). Reciprocal regulation among and miR395 posttranscriptionally APS and SULTR2; 1 *Arabidopsis thaliana*. *Plant Signal. Behav.* 5 1257–1259 10.1111/j.1365-313X 20935495 PMC3115361

[B40] Liu T. Y. Chang C. Y. Chiou T. J. (2009). The long-distance signaling of mineral macronutrients. *Curr. Opin. Plant Biol.* 12 312–319 10.1016/j.pbi.2009.04.004 19481493

[B41] Lu S. Sun Y. H. Chiang V. L. (2008). Stress-responsive microRNAs in Populus. *Plant J.* 55 131–151 10.1111/j.1365-313X.2008.03497.x 18363789

[B42] Lundmark M. Korner C. J. Nielsen T. H. (2010). Global analysis of microRNA in *Arabidopsis* in response to phosphate starvation as studied by locked nucleic acid-based microarrays. *Physiol. Plant.* 140 57–68 10.1111/j.1399-3054.2010.01384.x 20487378

[B43] Marín-González E. Suárez-López P. (2012). “And yet it moves”: cell-to-cell and long-distance signaling by plant microRNAs. *Plant Sci.* 196 18–30 10.1016/j.plantsci.2012.07.009 23017896

[B44] Martin A. Adam H. Diaz-Mendoza M. Zurczak M. Gonzalez-Schain N. Suarez-Lopez P. (2009). Graft-transmissible induction of potato tuberization by the microRNA miR172. *Development* 136 2873–2881. 19666819 10.1242/dev.031658

[B45] Matthewman C. A. Kawashima C. G. Huska D. Csorba T. Dalmay T. Kopriva S. (2012). miR395 is a general component of the sulfate assimilation regulatory network in *Arabidopsis*. *FEBS Lett.* 586 3242–3248 10.1016/j.febslet.2012.06.044 22771787

[B46] Nischal L. Mohsin M. Khan I. Kardam H. Wadhwa A. Abrol Y. P. (2012). Identification and comparative analysis of microRNAs associated with low-N tolerance in rice genotypes. *PLoS ONE* 7:e50261 10.1371/journal.pone.0050261 PMC3515565 23227161

[B47] Pant B. D. Buhtz A. Kehr J. Scheible W. R. (2008). MicroRNA399 is a long-distance signal for the regulation of plant phosphate homeostasis. *Plant J.* 53 731–738 10.1111/j.1365-313X.2007.03363.x 17988220 PMC2268993

[B48] Pant B. D. Musialak-lange M. Nuc P. May P. Buhtz A. Kehr J. (2009). Identification of nutrient-responsive *Arabidopsis* and rapeseed microRNAs by comprehensive real-time polymerase chain reaction profiling and small RNA sequencing. *Plant Physiol.* 150 1541–1555 10.1104/pp.109.139139 19465578 PMC2705054

[B49] Paul S. Ali N. Sarkar S. N. Datta S. K. Datta K. (2013). Loading and bioavailability of iron in cereal grains. *Plant Cell Tiss. Organ Cult.* 113 363–373 10.1007/s11240-012-0286-7

[B50] Pei L. Jin Z. Li K. Yin H. Wang J. Yang A. (2013). Identification and comparative analysis of low phosphate tolerance associated microRNAs in two maize genotypes. *Plant Physiol. Biochem.* 70 221–234 10.1016/j.plaphy.2013.05.043 23792878

[B51] Poirier Y. Bucher M. (2002). “Phosphate transport and homeostasis in *Arabidopsis,*” *in the Arabidopsis* eds Somerville R., Meyerowitz E. M.(Rockvilli, MD: American Society of Plant Biologists) 1 10.1199/tab.0024 PMC3243343 22303200

[B52] Rausch T. Wachter A. (2005). Sulfur metabolism: a versatile platform for launching defence operations. *Trends Plant Sci.* 10 503–509 10.1016/j.tplants.2005.08.006 16143557

[B53] Schauer S. E. Jacobson S. E. Meinke D. W. Ray A. (2002). DICER-LIKE1: blind men and elephants in *Arabidopsis* development. *Trends Plant Sci.* 7 487–491 10.1016/S1360-1385(02)02355-5 12417148

[B54] Sperotto R. A. Ricachenevsky F. K. Williams L. E. Vasconcelos M. W. Menguer P. K. (2014). From soil to seed: micronutrient movement into and within the plant. *Front. Plant Sci.* 5:438 10.3389/fpls.2014.00438 PMC4155779 25250035

[B55] Sunkar R. Kapoor A. Zhu J. K. (2006). Posttranscriptional induction of two Cu/Zn superoxide dismutase genes in *Arabidopsis* is mediated by downregulation of miR398 and important for oxidative stress tolerance. *Plant Cell* 18 2051–2065 10.1105/tpc.106.041673 16861386 PMC1533975

[B56] Sunkar R. Zhu J. K. (2004). Novel and stress-regulated microRNAs and other small RNAs from *Arabidopsis*. *Plant Cell* 16 2001–2019. 10.1105/tpc.104.022830 15258262 10.1105/tpc.104.022830 PMC519194

[B57] Valdes-Lopez O. Yang S. S. Aparicio-Fabre R. Graham P. H. Reyes J. L. Vance C. P. (2010). MicroRNA expression profile in common bean (*Phaseolus vulgaris*) under nutrient deficiency stresses and manganese toxicity. *New Phytol.* 187 805–818 10.1111/j.1469-8137.2010.03320.x 20553393

[B58] Varkonyi-Gasic E. Gould N. Sandanayaka M. Sutherland P. MacDiarmid R. M. (2010). Characterisation of microRNAs from apple (*Malus domestica* ‘Royal Gala’) vascular tissue and phloem sap. *BMC Plant Biol.* 10:159 10.1186/1471-222910-159 PMC3095296 20682080

[B59] Vazquez F. Legrand S. Windels D. (2010). The biosynthetic pathways and biological scopes of plant small RNAs. *Trends Plant Sci.* 15 337–345 10.1016/j.tplants.2010.04.001 20427224

[B60] Vigani G. Morandini P. Murgia I. (2013a). Searching iron sensors in plants by exploring the link among 2′-OG-dependent dioxygenases, the iron deficiency response and metabolic adjustments occurring under iron deficiency. *Front. Plant Sci.* 4:169 10.3389/fpls.2013.00169 PMC3668137 23755060

[B61] Vigani G. Zochhi G. Bashir K. Phillipar K. Briat J. F. (2013b). Cellular iron homeostasis and metabolism in plants. *Front. Plant Sci.* 4:490 10.3389/fpls.2013.00490 PMC3847546 24348493

[B62] Wang C. Huang W. Ying Y. Li S. Secco D. Tyerman S. (2012). Functional characterization of the rice SPXMFS family reveals a key role of OsSPX-MFS1 in controlling phosphate homeostasis in leaves. *New Phytol.* 196 139–148 10.1111/j.1469-8137.2012.04227.x 22803610

[B63] Wang M. Gruissem W. Bhullar N. K. (2013a). Nicotianamine synthase overexpression positively modulates iron homeostasis-related genes in high iron rice. *Front. Plant Sci.* 4:156 10.3389/fpls.2013.00156 PMC3665926 23755054

[B64] Wang Y. Zhang C. Hao Q. Sha A. Zhou R. Zhou X. (2013b). Elucidation of miRNAs-mediated responses to low nitrogen stress by deep sequencing of two soybean genotypes. *PLoS ONE* 8:e67423 10.1371/journal.pone.0067423 PMC3704600 23861762

[B65] Waters B. M. Mcinturf S. A. Stein R. J. (2012). Rosette iron deficiency transcript and microRNA profiling reveals links between copper and iron homeostasis in *Arabidopsis thaliana*. *J. Exp. Bot.* 63 5903–5918 10.1093/jxb/ers239 22962679 PMC3467300

[B66] Xu F. Liu Q. Chen L. Kuang J. Walk T. Wang J. (2013). Genome-wide identification of soybean microRNAs andtheir targets reveals their organ-specificity and responses to phosphate starvation. *BMC Genomics* 14:66 10.1186/1471-2164-14-66 PMC3673897 23368765

[B67] Xu Z. Zhong S. Li X. Li W. Rothstein S. J. Zhang S. (2011). Genome-wide identification of microRNAs in response to low nitrate availability in maize leaves and roots. *PLoS ONE* 6:e28009 10.1371/journal.pone.0028009 PMC3223196 22132192

[B68] Yan Z. Hossain M. S. Wang J. Valdes-Lopez O. Liang Y. Libault M. (2013). miR172 regulates soybean nodulation. *Mol. Plant Microbe Interact.* 26 1371–1377 10.1094/MPMI-04-13-0111-R 23980625

[B69] Zeng H. Q. Zhu Y. Y. Huang S. Q. Yang Z. M. (2010). Analysis of phosphorus-deficient responsive miRNAs and cis elements from soybean (*Glycine max* L.)*. J. Plant Physiol.* 167 1289–1297 10.1016/j.jplph.2010.04.017 20591534

[B70] Zeng Q. Y. Yang C. Y. Ma Q. B. Li X. P. Dong W. W. Nian H. (2012). Identification of wild soybean miRNAs and their target genes responsive to aluminum stress. *BMC Plant Biol.* 12:182 10.1186/1471-2229-12-182 PMC3519564 23040172

[B71] Zhang B. Pan X. Stellwag E. J. (2008). Identification of soybean microRNAs and their targets. *Planta* 229 161–182 10.1007/s00425-008-0818-x 18815805

[B72] Zhang L. W. Song J. B. Shu X. X. Zhang Y. Yang Z. M. (2013). miR395 is involved in detoxification of cadmium in *Brassica napus*. *J. Hazard Mater.* 250–251, 204–211 10.1016/j.jhazmat.2013.01.053 23454459

[B73] Zhao M. Ding H. Zhu J. K. Zhang F. Li W. X. (2011). Involvement of miR169 in the nitrogen-starvation responses in *Arabidopsis*. *New Phytol.* 190 906–915 10.1111/j.1469-8137.2011.03647.x 21348874 PMC3586203

[B74] Zhao M. Tai H. Sun S. Zhang F. Xu Y. Li W. X. (2012). Cloning and characterization of maize miRNAs involved in responses to nitrogen deficiency. *PLoS ONE* 7:e29669 10.1371/journal.pone.0029669 PMC3250470 22235323

[B75] Zhao X. Liu X. Guo C. Gu J. Xiao K. (2013a). Identification and characterization of microRNAs from wheat (*Triticum aestivum* L.) under phosphorus deprivation. *J. Plant Biochem. Biotechnol.* 22 113–123 10.1007/s13562-012-0117-2

[B76] Zhao Y. Xu Z. Mo Q. Zou C. Li W. Xu Y. (2013b). Combined small RNA and degradome sequencing reveals novel miRNAs and their targets in response to low nitrate availability in maize. *Ann. Bot.* 112 633–642 10.1093/aob/mct133 23788746 PMC3718221

[B77] Zhou Z. S. Song J. B. Yang Z. M. (2012). Genome-wide identification of *Brassica napus* microRNA and their targets in response to cadmium. *J. Exp. Bot*. 63 4597–4613 10.1093/jxb/ers136 22760473 PMC3421990

[B78] Zhu Y. Y. Zeng H. Q. Dong C. X. Yin X. M. Shen Q. R. Yang Z. M. (2010). MicroRNA expression profiles associated with phosphorus deficiency in white lupin (*Lupinus albus* L.). *Plant Sci.* 178 23–29 10.1016/j.plantsci.2009.09.011

